# Loss of cadherin related family member 5 (CDHR5) expression in clear cell renal cell carcinoma is a prognostic marker of disease progression

**DOI:** 10.18632/oncotarget.20507

**Published:** 2017-08-24

**Authors:** Felix Marius Bläsius, Sebastian Meller, Carsten Stephan, Klaus Jung, Jörg Ellinger, Michael O. Glocker, Hans-Jürgen Thiesen, Yuri Tolkach, Glen Kristiansen

**Affiliations:** ^1^ Institute of Pathology, University Hospital Bonn, Bonn, Germany; ^2^ Clinic of Urology, Charité-Universitätsmedizin Berlin, Berlin, Germany; ^3^ Berlin Institute for Urologic Research, Robert-Koch Platz 7, Berlin, Germany; ^4^ Clinic of Urology, University Hospital Bonn, Bonn, Germany; ^5^ Proteome Center Rostock, University of Rostock, Rostock, Germany

**Keywords:** CDHR5, renal cell carcinoma, immunohistochemistry, prognostic marker

## Abstract

Reduced expression of Cadherin-Related Family Member 5 (CDHR5) was recently found implied in carcinogenesis of colon cancer, but its role in other tumors is unknown. We aimed to analyze the expression of CDHR5 in different subtypes of renal cell carcinoma. CDHR5 expression was immunohistochemically examined using tissue micro arrays (TMAs) covering 279 patients with primary renal cell carcinoma. Additionally, expression data from the TCGA (The Cancer Genome Atlas) of an independent cohort of 489 clear-cell RCC cases was evaluated. CDHR5 protein expression was found in 74.9% of cases, with higher levels seen in clear cell and papillary RCC. In the univariate analysis CDHR5 expression was significantly associated with a longer overall survival of RCC patients at the protein (p = 0.026, HR = 0.56) and transcript levels (TCGA-cohort: p = 0.0002, HR = 0.55). Importantly, differences in survival times were confirmed independently in multivariate analyses in a model with common clinicopathological variables at the transcript level (p = 0.0097, HR = 0.65). Investigation of the putative functional role of CDHR5 using TCGA data and Enrichment analysis for Gene Ontology and Pathways revealed associations with many metabolic and some tumor growth-associated processes and pathways. CDHR5 expression appears to be a promising and new independent prognostic biomarker in renal cell carcinoma.

## INTRODUCTION

In Germany, renal cell carcinoma (RCC) is the 8^th^ most common cancer type in men and 10^th^ most in women with 30,400 new cases and 10,500 deaths estimated for 2012 in Germany referring to a mortality of about 34% [1]. From 2000 to 2010 the cancer incidence increased by almost 10%. The majority of patients present at the time of initial diagnosis with relatively small tumors that are treated by radical nephrectomy or, as Van Poppel *et al.* [2] showed for low-stage RCC, by nephron-sparing surgery. However, 33% of the patients show symptomatic metastases at the time of diagnosis [3]. Furthermore, about 40% of the patients will develop metastasis or recurrences after nephrectomy [4, 5]. The 5-year relative survival rates range from 75% for men to 77% for women [6].

Metastatic RCC shows low response rates to standard chemotherapy. The management of advanced RCC has dramatically changed over the past decade. New multitargeted cancer therapies demonstrated improved response rates and a survival benefit for the patient, however, most patients with advanced RCC still have limited overall survival [7]. The large number of pathways, which are changed in tumor cells, open up the possibility for targeted new treatment options [8, 9].

Concerning this field of research, cell adhesion molecules, such as cadherin superfamily might be key players in developmental processes regarding to cell segregation and tumorigenesis [10, 11]. For some members of cadherin superfamily like E-cadherin and N-cadherin numerous clinical studies have shown that decreased expression of E-cadherin and thus overexpression of N-Cadherin promote motility and invasion [12]. The influence of cadherin’s could be shown for various tumor entities, including hereditary gastric cancer [13], breast cancer [14, 15] and renal cell carcinoma [16].

We decided to perform a further evaluation of the Cadherin-Related Family Member 5 (CDHR5), which is a protocadherin genomically located in the 11p15.5 chromosome region. Aberrations in this region are also associated with the Beckwith-Wiedmann syndrome and show as a second locus, a loss of heterozygosity in Wilms tumors [17-19]. Moreover CDHR5 is a protocadherin with a characteristic mucin-type repeat structure and two isoforms [20]. It is expressed in the basolateral membrane of epithelial structures during kidney and lung development [21]. CDHR5 plays an important role in brush border assembly in intestinal epithelium [22]. As it is reported for the cadherin superfamily, CDHR5 is a Ca^2+^-dependent cell-cell adhesion molecule including a possible role as a receptor for extracellular signals [23].

We aimed to assess the expression of CDHR5 in benign renal tissues and RCC and correlate these findings to clinicopathologic parameters including patient survival in two independent cohorts (Charité-Universitätsmedizin Berlin, Germany and the RCC cohort of The Cancer Genome Atlas (TCGA; http://cancergenome.nih.gov/).

## RESULTS

### CDHR5 expression in renal and cancerous tissues

In normal renal tissues, CDHR5 was immunohistochemically detected in the proximal tubule with a pronunciation at the luminal brush border. The glomeruli, the distal tubuli, the collecting ducts and the stromal cells were all negative.

**Figure 1:**
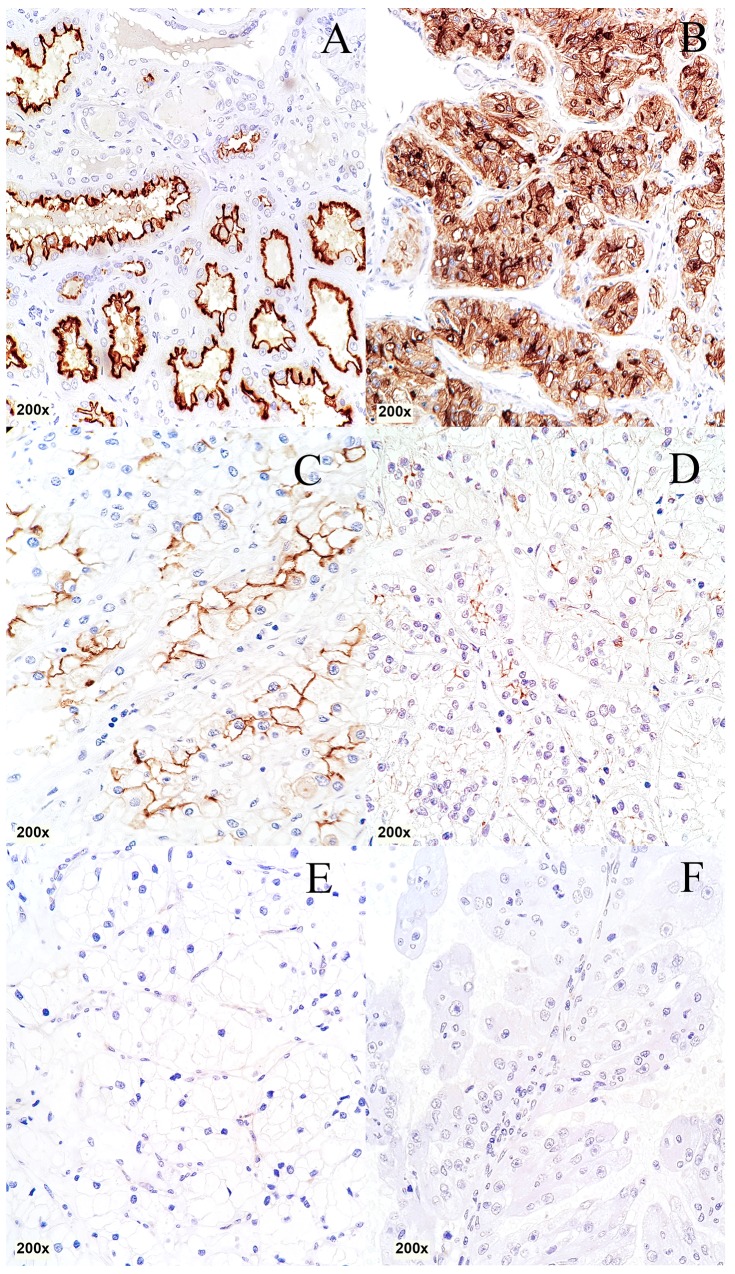
Immunohistochemical staining of CDHR5 in different histological subtypes of RCC **(A)** Normal kidney, **(B)** clear cell RCC: positive “3+”, **(C)** clear cell RCC: positive “2+”, **(D)** clear cell RCC: positive “1+”, **(E)** clear cell RCC: negative “0”, **(F)**: papillary RCC: negative “0”. (total magnification for every image 200x)

Among the cancerous tissue no CDHR5 expression could be observed in 70 of 279 evaluable cases (25.1%), whereas 125/279 (44.8%) showed a weak, 66/279 (23.7%) revealed a moderate and 18/279 (6.5%) showed a strong CDHR5 immunoreactivity. Representative immunostainings of CDHR5 in RCC are given in Figure 1.

A stratified analysis of CDHR5 expression according to histological tumor subtype showed, that CDHR5 is mostly expressed in clear cell and papillary RCC (77.3% and 78.1% positive cases, respectively) whereas chromophobe RCC (n=9) showed no expression at all (Table 1).

**Table 1:** Associations of CDHR5 expression with clinicopathological parameters of RCC patients

	All patients (n=279)	CDHR5-negative	CDHR5-positive	p-Value
Median follow-up [months]	87 (0-177)			
**Evaluable cases**	279	70 (25.1%)	209 (74.9%)	
**Age, mean (range/SD)**	62 (30-86)	61.0±11.4	61.2±9.4	0.89
**Gender**				0.049*
Men	190 (68.1%)	41 (21.6%)	149 (78.4%)	
Women	89 (31.9%)	29 (32.6%)	60 (67.4%)	
**Histology**				0.918**
Clear cell	238 (85.3%)	54 (22.7%)	184 (77.3%)	
Papillary	32 (11.5%)	7 (21.9%)	25 (78.1%)	
Chromophobe	9 (3.2%)	9 (100.0%)	0 (0%)	
**pT status**				0.003^$^
pT_1_	158 (56.6%)	26 (16.5%)	132 (83.5%)	
pT_2_	25 (9.0%)	10 (40.0%)	15 (60.0%)	
pT_3a_	37 (13.2%)	11 (29.7%)	26 (70.3%)	
pT_3b_	53 (19.0%)	20 (37.7%)	33 (62.3%)	
pT_3c_/pT_4_	6 (2.2%)	3 (50.0%)	3 (50.0%)	
**pN status**				0.305*
pN_0_	144 (51.6%)	37 (25.7%)	107 (74.3%)	
pN_1_	19 (6.8%)	7 (36.8%)	12 (63.2%)	
pNx	116 (41.6%)			
**ISUP grade**				0.419^$^
G_1_	44 (16.3%)	10 (22.7%)	34 (77.3%)	
G_2_	152 (56.3%)	33 (21.7%)	119 (78.3%)	
G_3_	59 (21.9%)	12 (20.3%)	47 (79.7%)	
G_4_	15 (5.6%)	6 (40.0%)	9 (60.0%)	
No grade (chromophobe)	9			
**Metastasis**				-
M_0/x_	243 (87.1%)	59 (24.3%)	184 (75.7%)	
M_1_ synchronic	25 (9.0%)	7 (28.0%)	18 (72.0%)	
M_1_ asynchronic	11 (3.9%)	4 (36.4%)	7 (63.6%)	
**ECOG performance status**				0.166^$^
0	189 (67.7%)	43 (22.8%)	146 (77.2%)	
1	81 (29.0%)	26 (32.1%)	55 (67.9%)	
2	9 (3.2%)	1 (11.1%)	8 (88.9%)	

* Mann-Whitney, ** Mann-Whitney for Clear cell vs Papillary histology, $ - Kruskall-Wallis-Test, SD – standard deviation.

In 100 (35.8%) of the 279 RCC tumor samples CDHR5 was expressed solely luminal. In the remaining cases the CDHR5 expression was diffuse or lost. In two thirds (157 of 238) of the clear-cell RCC cases, CDHR5 was expressed not only luminally, whereas, in 59.4% of the papillary RCCs (19 of 32) CDHR5 was exclusively expressed at the luminal brush border.

### Association of CDHR5 expression with clinicopathological parameters and overall survival

Tissue samples from 279 RCC patients were analyzed (immunohistochemistry). A weak correlation between CDHR5 expression and the pT-stage (r = -0.143, p = 0.005) was found. A higher percentage of men showed CDHR5 expression with on average higher CDHR5 levels compared to women (p = 0.049). No significant association could be drawn between CDHR5 expression and (I) the pN-, M- and R-staging (II) patient age and (III) the ISUP Grade (Table 1).

**Figure 2:**
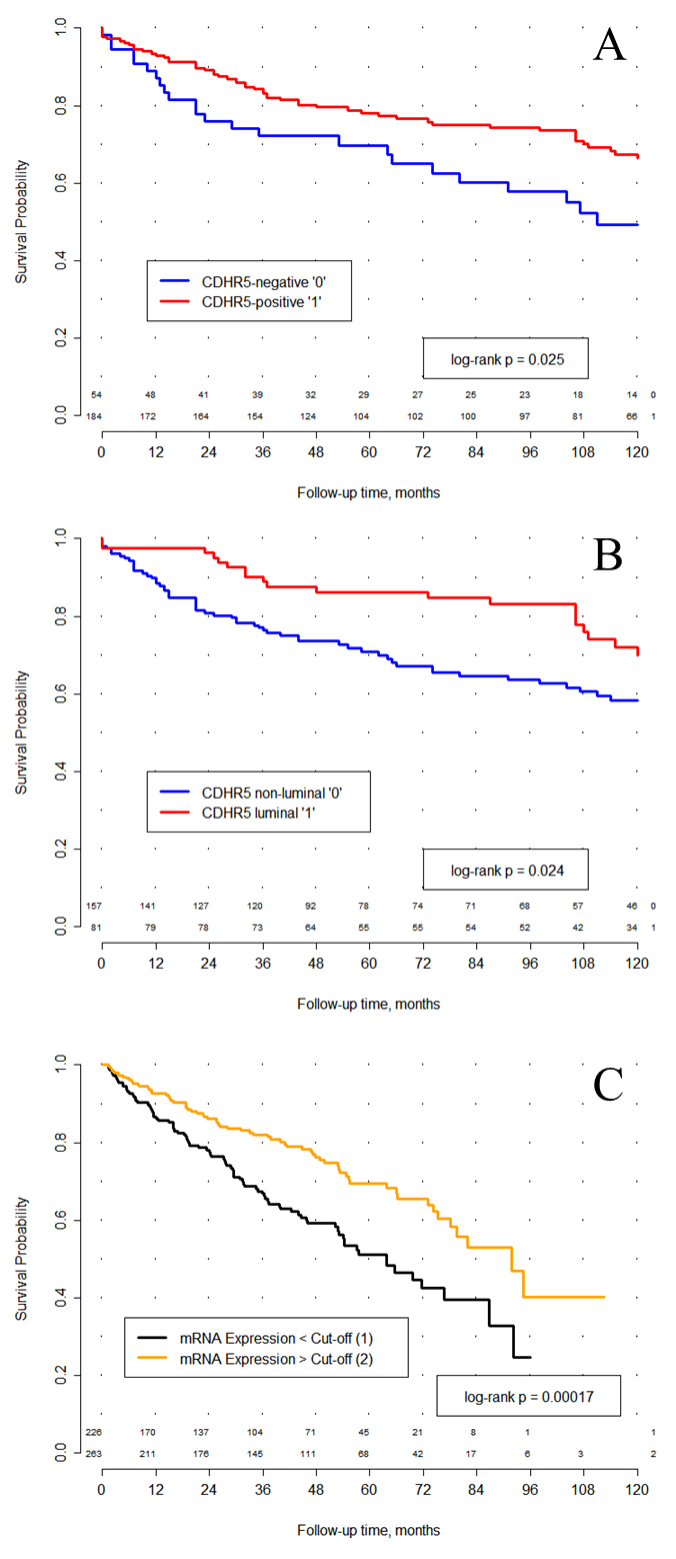
Kaplan-Meier analysis of overall survival **(A)** In a cohort comprised of 238 renal cell carcinoma (RCC) patients with clear cell histology a significant association between CDHR5 expression and overall survival after surgery was observed. CDHR5 expression was dichotomized into CDHR5-negative (n = 54) vs. CDHR5-positive (n = 184), based on the complete absence or presence of any immunohistochemical protein expression. **(B)** CDHR5 expression was dichotomized into luminal CDHR5 expression (n = 81) vs. diffuse or no CDHR5 expression (n = 157). In a cohort comprised of 238 clear-cell RCC cases, patients with luminal CDHR5 expression showed a significant longer overall survival after surgery compared to those with no or diffuse CDHR5 expression. **(C)** In 489 RCC patient samples from the TCGA cohort the prognostic value of *CDHR5* expression could be confirmed (log rank p = 0.00017). *CDHR5* expression values (RNASeq) were dichotomized based on the best cut-off.

For survival analyses we have used only the patients with clear cell histology (ccRCC) of RCC (n=238) to enable further comparisons to TCGA-data. CDHR5 expression was dichotomized into CDHR5-negative (n = 54) vs. CDHR5-positive (n = 184) cases. In a univariate Cox proportional Hazard analysis CDHR5-positive ccRCC patients showed a significantly lower risk of death compared to the CDHR5-negative cases (p = 0.027, HR = 0.58, CI 95% [0.358-0.939]). This finding could be confirmed in a Kaplan-Meier analysis (p = 0.025, Figure 2A). In a multivariate survival analysis with inclusion of the pT- and pN-stage, R-status and ISUP grading CDHR5 expression (immunohistochemistry) demonstrated no independent prognostic value (Table 2). Similar results for univariate and multivariate analyses were received in analysis with inclusion of clear cell and papillary histological types of RCC together (data not shown).

**Table 2:** Univariate and multivariate Cox proportional hazard analyses on overall survival in a sub-cohort of clear-cell renal cell carcinoma patients (n=238)

Clinicopathological parameter / biomarker	Univariate Cox		Multivariate Cox	
	Hazard ratio [95% CI]	p-Value	Hazard ratio [95% CI]	p-Value
Tumor stage:				
pT1	1.00	-	1.00	-
pT2	2.69 [1.14-6-36]	0.024	1.72 [0.68-4.38]	0.255
pT3+pT4	5.77 [3.43-9.72]	<0.001	4.07 [2.29-7.21]	<0.001
ISUP grade:				
Grade 1	1.00	-	1.00	-
Grade 2	3.05 [1.21-7.71]	0.018	2.35 [0.92-6.04]	0.075
Grade 3	4.36 [1.64-11.56]	0.003	3.15 [1.16-8.54]	0.024
Grade 4	9.48 [3.17-28.34]	<0.001	4.34 [1.36-13.85]	0.013
Surgical margin (R1 vs. R0)	7.64 [3.96-14.75]	<0.001	3.25 [1.63-6.45]	0.001
Nodal status (pN1 vs. pN0/x)	3.35 [1.72-6.52]	<0.001	1.25 [0.58-2.68]	0.558
CDHR5 immunohistochemistry (negative vs positive)	1.73 [1.07-2.80]	0.027	1.36 [0.81-2.26]	0.243
CDHR5 immunohistochemistry (luminal vs non luminal)	0.56 [0.34-0.93]	0.026	#	#

^#^CDHR5 luminal vs non luminal was not significant in the multivariate Cox proportional hazard analysis in the model with the same list of parameters.

Furthermore, the effect of the luminal limited CDHR5-expression on the prognosis of the RCC-patients (clear-cell and papillary) and on the correlation with clinicopathological parameters was examined. As described for the CDHR5-expression-strength a correlation between the luminal localization of the CDHR5-expression and the T-stage (r = -0.209, p = 0.001) could be found. No correlation between luminal expression of CDHR5 and the tumor grade (ISUP grading) was observed. Patients with tumors that at least partially comprise a tubulus like CDRH5-positivity had a favorable prognosis compared to those patients where the CDHR5-expression in the tumors is lost or not luminal restricted (p = 0.009, HR = 0.53, CI 95% [0.33-0.85]). This finding could be confirmed in a Kaplan-Meier analysis (p = 0.007, Figure 2B)

### Analysis of prognostic role and investigation of putative biological role (TCGA-data)

Results from survival analysis were validated in an independent testing cohort comprised of clear cell RCC patients included in TCGA. A best cut-off for transcript number, based on the cut-off analysis (2580.166), classified patients approximately in the middle of the cohort into m*CDHR5*-low (n = 226), and m*CDHR5-*high (n = 263) groups. In univariate Cox proportional hazards analysis high *CDHR5* mRNA expression (as assessed by RNASeq) showed a significantly lower risk of earlier death compared to patients with low *CDHR5* mRNA expression (p = 0.0002, HR = 0.55, 95% CI [0.40-0.75]; Table 3). This finding was confirmed in a Kaplan-Meier analysis of overall survival (log rank p = 0.0002, Figure 2C).

**Table 3:** Univariate and multivariate Cox proportional hazard analyses on overall survival of clear-cell renal cell carcinoma patients from the TCGA cohort (n=489)

Clinicopathological parameter / biomarker^#^	Univariate Cox		Multivariate Cox	
	Hazard ratio [95% CI]	p-Value	Hazard ratio [95% CI]	p-Value
Tumor stage				
pT1	1.00	-	1.00	-
pT2	1.44 [0.82-2.52]	0.202	1.28 [0.72-2.26]	0.401
pT3a	3.61 [2.45-5.33]	9.68e-11	2.41 [1.58-3.69]	4.68e-05
pT3b	3.46 [2.10-5.70]	1.14e-06	2.57 [1.53-4.31]	0.0004
pT3c	8.22 [1.98-34.13]	0.004	4.55 [1.07-19.37]	0.040
pT4	12.55 [6.29-25.07]	7.55e-13	4.64 [1.83-11.78]	0.001
ISUP grade				
Grade 2	1.00	-	1.00	-
Grade 3	1.88 [1.26-2.81]	0.002	1.48 [0.98-2.24]	0.060
Grade 4	5.67 [3.72-8.66]	8.88e-16	2.83 [1.73-4.62]	3.33e-05
Nodal status (pN1 vs. pN0/x)	3.75 [1.97-7.13]	5.72e-05	1.12 [0.49-2.78]	0.729
*CDHR5* mRNA Expression (high vs low*)	0.55 [0.40-0.75]	0.0002	0.65 [0.47-0.90]	0.0097

^#^ Surgical margin status was not available. * “high” means > cut-off, “low” means < cut-off.

A multivariate Cox proportional hazards of the *CDHR5* mRNA expression using selected cut-off in the model including ISUP grade, pT-, pN-stage and R-Status showed an independent significant prognostic value (p = 0.0097, HR = 0.65, 95% CI [0.47-0.90]; Table 3).

The enrichment analysis for Gene Ontology and Pathways as an investigation tool for putative biological role of CDHR5 have shown the associations of this gene with important metabolic pathways (including many interactions with genes related to transmembrane and intracellular transport of different substances) and also with pathways related to cell motion, cell migration, apoptosis induction and signal transduction (Supplementary Figures 1 and 2).

## DISCUSSION

In this study we showed for the first time CDHR5 expression in a population based RCC patient cohort. CDHR5 positivity is significantly associated with a longer overall survival time. The association between CDHR5 expression and RCC patient survival at the transcript level (TCGA RNAseq data) remained significant after multivariable adjustment for other clinicopathological parameters, including pT-, pN- categories, surgical margin status and grade. These results were confirmed in an independent RCC patient cohort from TCGA. Thus, the association appeared to be independent of current prognostic factors and could offer additional relevant prognostic information.

In the kidney, CDHR5 is primarily located at the luminal brush border in proximal tubules. So far, the physiological functions of CDHR5 are not sufficiently characterized. Similar to classical cadherins it is calcium ion binding and also β-catenin binding [24]. As a member of cadherin superfamily it can be assumed that CDHR5 mediates cell-cell adhesion and cell-signalling. In fact, specific function in normal cells is not yet described. In relation to pathogenesis CDHR5 influence is described for gallstone disease [25] and systemic sclerosis [26]. In addition to that, *CDHR5* promoter methylation is a possible prognostic biomarker for cyst growth in polycystic kidney disease [27].

In tumors CDHR5 expression is altered. Recent studies showed that CDHR5 downregulation is a common event in colorectal carcinoma cells [28, 29]. In contrast, mesalazin therapy leads to a growth arrest due to higher CDHR5 expression in colorectal carcinoma (CRC) by inducing an increased transcription of the cyclin-dependent kinase inhibitor (p21^waf-1^) gene [24]. Besides the Cdx2 Homeobox, which regulates the balance between proliferation and differentiation in the adult intestinal epithelium, it also regulates CDHR5 expression by binding to two consensus Cdx2-binding sites. In CRC cell lines, expression of CDHR5 leads to a reduction of colonies and a disbanding of established colonies [30]. A possible mechanism is the ability of CDHR5 to retain β-catenin on the plasmatic membrane in CRC tumor cells. β-catenin acts as a transcription factor and is normally controlled by degradation through APC, which in turn can be inhibited by Wnt signalling. Therefore, CDHR5 has a direct influence on Wnt/β-catenin pathway, which is considered important in CRC tumorigenesis [31]. It can be assumed that catenins regulate gene expression and that these proteins form a functional network in the nucleus [32]. Former studies have indicated that the Wnt/β-catenin pathway may also be relevant in renal carcinogenesis [33]. Especially, the dysregulation of β-catenin in ccRCC is a predictor of a lower tumor mortality [34] and a potential target for therapy [35].

The ability of CDHR5 to retain β-catenin at the plasma membrane and to influence Wnt signaling is therefore consistent with the results of our study, as CDHR5 expression correlates with longer overall survival times of RCC patients in general. This was particularly pronounced in cases with a strict luminal localization of CDHR5 on the luminal brush border. CDHR5 expression was especially observed in clear cell and papillary RCCs which supports the notion that these subtypes share molecular associations with epithelia from proximal tubules [36] and that a correct expression of CDHR5 is important for the microvilli and epithelial brush border differentiation [22]. CDHR5 localization showed, in contrast to expression, significantly more reliable judgment about the overall patient survival.

Our analysis of the putative functional role of the CDHR5 based on the Enrichment analysis for Gene Ontology and Pathways provided some interesting findings, which certainly need to be proved in further functional studies, namely, that CDHR5 seems to exercise its main functions in the sphere of metabolic processes, although some important tumor growth-associated processes / pathways could be also linked to this gene.

A few limitations of the study need to be mentioned. A major limitation of this retrospective study is the relatively low number of patients with a non-clear cell histology, which are not sufficiently explored by our study. The role of cell adhesion molecules such as the cadherins in renal carcinogenesis ought to be explored further. This study is only descriptive and does not provide any functional analyses of CDHR5 in RCC cells. Further studies need to provide information whether CDHR5 has a comparable function in renal cell carcinogenesis like in CRC carcinogenesis.

In summary, this is the first study, which describes CDHR5 expression in RCC. CDHR5 loss appears to be a promising prognostic biomarker for clear cell renal cell carcinoma patients. The functional role of CDHR5 and its expression patterns in other renal tumor entities need to be unraveled in future studies.

## MATERIALS AND METHODS

### Ethics statement

The studies were approved by the Institutional Review Board (IRB) Charite - Universitätsmedizin Berlin (EA1/06/2004).

### Patients and tissue microarray construction

The patient cohort and the construction of the tissue microarray were described in former studies [37]. Our cohort consists of two hundred seventy-nine primary RCC cases which were staged with regard to International Union against Cancer 2002 criteria at the Institute of Pathology, Charité-Universitätsmedizin Berlin. Patients underwent surgery at the Department of Urology, Charité-Universitätsmedizin Berlin, between 1993 and 2004. 190 (68.1%) of the patients were male and 89 were female (31.9%). The mean age at the time of surgery was 62 years (range 30-86). The dominating histological subtype was clear cell RCC (n = 238, 85.3%), papillary RCC were less common (n=32, 11.5%), as well as chromophobe RCC were rare (n = 9, 3.2%). All case were subjected to a central review to adjust the tumor grade according to the recommendations of ISUP 2012/WHO 2016 (YT, GK). Clinical characteristics are outlines in the Table 1.

For all 279 cases follow-up data were available. The median follow-up time was 87 months (range 0-177 months). In order to avoid contamination of progression data by age-related death we truncated follow-up times after 120 months. During this time 87 patients died (31.2%) after a median survival time of 31 months.

### Immunohistochemistry

The TMAs were stained in the immunohistochemistry laboratory at the Institute of Pathology, Bonn. We used the Lab Vision Autostainer 480S system (Thermo Scientific, Waltham, MA, USA) together with the Thermo Scientific Reagents and the Medac C-DPVB 500 HRP detection system. The PT-Module was used for dewaxing and epitope retrieval (pH 6.0 at 99°C for 20 min). The polyclonal anti-CDHR5 antibody HPA009173 (Sigma Aldrich) was used (dilution 1:250), which was specificity-validated by the Human Protein Atlas (www.proteinatlas.org/). Slides were counterstained with hematoxylin, dehydrated and aqueously mounted.

CDHR5 immunoreactivity was semi-quantitatively scored and categorised as follows: negative (0), weak (1), moderate (2) and strong (3). Furthermore, the pattern of CDHR5 immunoreactivity was recorded (luminal vs. diffuse or negative).

### TCGA-data mining

For validation of the results from the training study, an independent series of 489 ccRCC patients with follow-up (more than 30 days) and clinicopathological data was evaluated. The patients with ISUP Grade 1 (n=8) were not used in the univariate and multivariate analysis, as they showed a uniform excellent overall survival, which distorts the analysis when those patients are used as comparator. The results from the validation cohort shown here are in whole based upon RNASeq data generated by the TCGA Research Network: (http://cancergenome.nih.gov/). Normalized results of the mRNA expression were used for analysis. Overall survival was used as the end-point. The number of death events in the cohort was 158/489 (32.3%).

TCGA data set was also used for investigations of the functional role of CDHR5. Briefly, we have taken mRNA expression data of all genes evaluated using RNAseq (20501 genes) in tumor samples of patients with clear-cell RCC. Enrichment analysis for Gene Ontology and Pathways have been carried out using TCGABiolinks package for R to reveal the functions and to integrate the CDHR5 into certain pathways based on the analysis of associations with other genes. Two modifications of this analysis were performed. Firstly, we have analysed top 100 of genes, the mRNA expression of which was highly correlated with CDHR5 (Supplementary Tables 1 and 2), among them 50 genes with positive correlation and 50 genes with negative correlation. Secondly, we have performed the additional investigations for top 100 genes with differential expression (50 upregulated and 50 downregulated) between samples with high and low CDHR5 mRNA expression using the cut-off for transcript number from our prognostic studies (Supplementary Table 3), which proved to have the highest prognostic value with regard to survival and statistical significance (Cut-off = 2580.166).

### Statistics

Statistical analysis was performed with SPSS, Version 21 (IBM SPSS Statistic) and R (version 3.2.2). Fisher’s exact test and chi-square test were used to evaluate statistical significance of the associations between CDHR5 expression and clinicopathological parameters. Bivariate correlation analysis was carried out using Spearman’s Rho. Survival analyses were conducted according to uni- and multivariate Cox Proportional Hazards and Kaplan-Meier analyses. P values refer to Wald test and Log-rank test. An error value of 5% (α = 0.05) was defined to infer statistical significance. As we do not consider such long follow-up relevant for RCC-specific survival, follow-up was truncated after 10 years. Extraction of the TCGA mRNA expression data was done in R using TCGA-Biolinks package. The best cut-off for the mRNA expression on the TCGA data was selected in R using the survMisc package (automatized systematic univariate Cox regression-based analysis of all available cut-offs for mRNA expression of CDHR5 using overall survival as end-point).

## SUPPLEMENTARY MATERIALS FIGURES AND TABLES









## Abbreviations

ccRCC: clear cell renal cell carcinoma
HR: hazard ratio
ISUP: Internation Society of Urological Pathology
RCC: renal cell carcinoma
TCGA: The Cancer Genome Atlas
WHO: World Health Organization
