# Supplementary material for: Loss of cadherin related family member 5 (CDHR5) expression in clear cell renal cell carcinoma is a prognostic marker of disease progression
Source: Oncotarget. 2017 Aug 24;8(43):75076–86. doi: 10.18632/oncotarget.20507 (PMC5650401; doi:10.18632/oncotarget.20507)
Supplement: Supplementary file 1 [file oncotarget-08-75076-s001.pdf]

Loss of cadherin related family member 5 (CDHR5) expression in clear cell renal cell carcinoma is a prognostic marker of disease progression

SUPPLEMENTARY MATERIALS

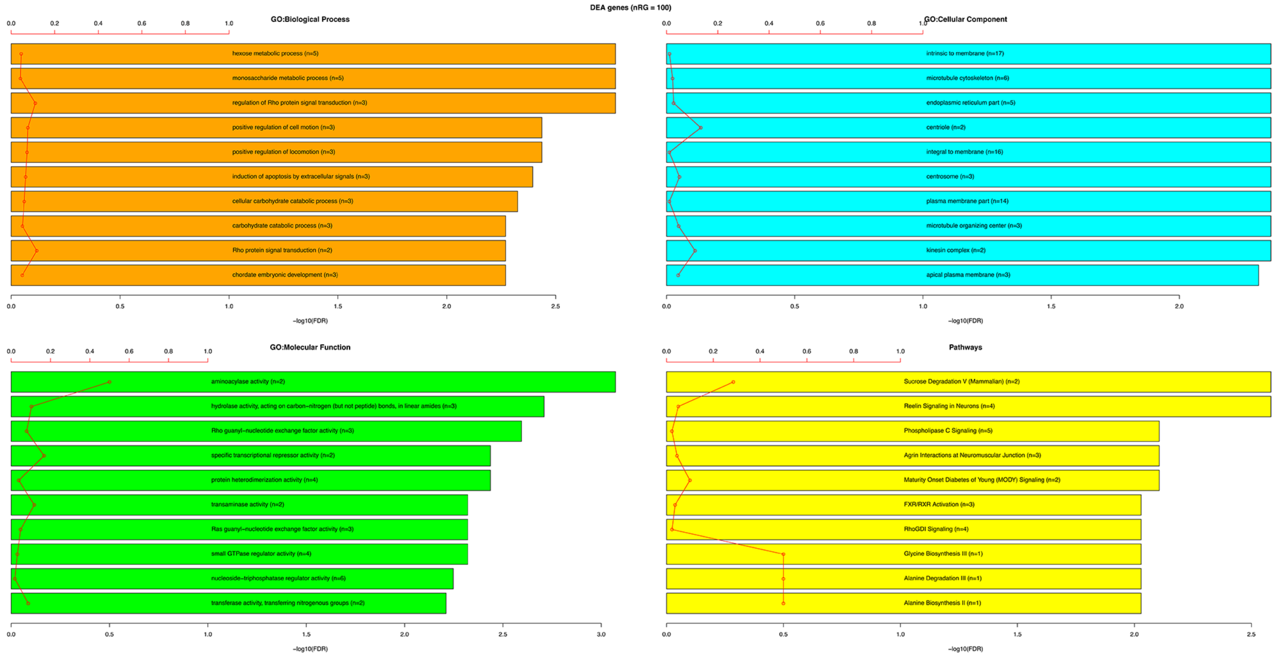

Supplementary Figure 1: Enrichment analysis for Gene Ontology and Pathways for Top 100 Genes associated with CDHR5 based on the correlation analysis for mRNA expression.

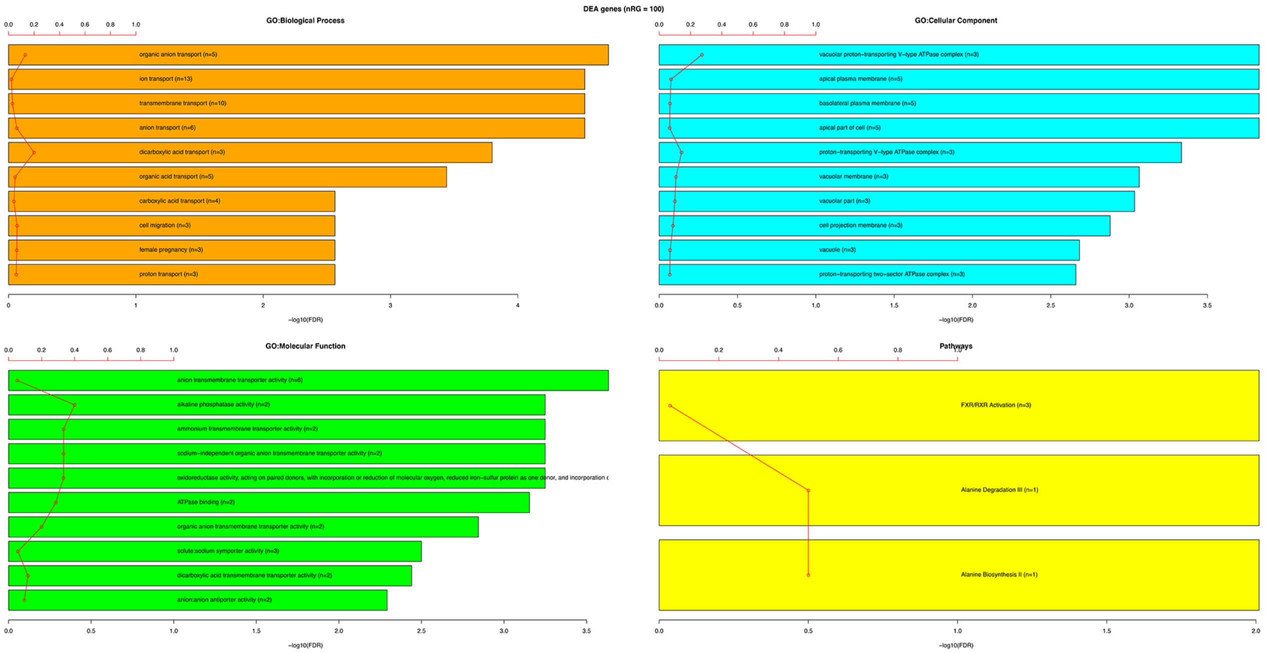

Supplementary Figure 2: Enrichment analysis for Gene Ontology and Pathways for Top 100 Genes with differential mRNA expression between tumor samples with high and low mRNA expression of CDHR5 (Cut-off for CDHR5 mRNA transcript number 2580.166).

Supplementary Table 1: Correlation between mRNA expression of CDHR5 with other genes (full list of genes).

See Supplementary File 1

Supplementary Table 2: Top 100 Genes, mRNA expression of which is highly correlated to mRNA expression of CDHR5.

See Supplementary File 2

Supplementary Table 3: Top 100 genes differentially expressed in tumor samples with high and low CDHR5 mRNA expression.

See Supplementary File 3
